# Psychometric properties of the mock interview rating scale for autistic transition-age youth

**DOI:** 10.3389/fpsyt.2023.1235056

**Published:** 2023-11-06

**Authors:** Matthew J. Smith, Kari L. Sherwood, Helen M. Genova, Brittany Ross, Leann Smith DaWalt, Lauren Bishop, David Telfer, Cheryl Brown, Barbara Sanchez, Michael A. Kallen

**Affiliations:** ^1^School of Social Work, University of Michigan, Ann Arbor, MI, United States; ^2^Department of Psychology, University of Michigan, Ann Arbor, MI, United States; ^3^Kessler Foundation, East Hanover, NJ, United States; ^4^Department of Physical Medicine and Rehabilitation, Rutgers New Jersey Medical School, Newark, NJ, United States; ^5^Waisman Center, University of Wisconsin, Madison, WI, United States; ^6^Sandra Rosenbaum School of Social Work, University of Wisconsin, Madison, WI, United States; ^7^Consultant, Warren, RI, United States; ^8^Ann Arbor Public Schools, Ann Arbor, MI, United States; ^9^Ann Arbor Academy, Ann Arbor, MI, United States; ^10^Feinberg School of Medicine, Northwestern University, Chicago, IL, United States

**Keywords:** autism, job interview skills assessment, psychometric properties, employment, transition-age youth

## Abstract

**Background:**

Employment is a major contributor to quality of life. However, autistic people are often unemployed and underemployed. One potential barrier to employment is the job interview. However, the availability of psychometrically-evaluated assessments of job interviewing skills is limited for autism services providers and researchers.

**Objective:**

We analyzed the psychometric properties of the Mock Interview Rating Scale that was adapted for research with autistic transition-age youth (A-MIRS; a comprehensive assessment of video-recorded job interview role-play scenarios using anchor-based ratings for 14 scripted job scenarios).

**Methods:**

Eighty-five transition-age youth with autism completed one of two randomized controlled trials to test the effectiveness of two interventions focused on job interview skills. All participants completed a single job interview role-play at pre-test that was scored by raters using the A-MIRS. We analyzed the structure of the A-MIRS using classical test theory, which involved conducting both exploratory and confirmatory factor analyzes, Rasch model analysis and calibration techniques. We then assessed internal consistency, inter-rater reliability, and test–retest reliability. Pearson correlations were used to assess the A-MIRS’ construct, convergent, divergent, criterion, and predictive validities by comparing it to demographic, clinical, cognitive, work history measures, and employment outcomes.

**Results:**

Results revealed an 11-item unidimensional construct with strong internal consistency, inter-rater reliability, and test–retest reliability. Construct [pragmatic social skills (*r* = 0.61, *p* < 0.001), self-reported interview skills (*r* = 0.34, *p* = 0.001)], divergent [e.g., age (*r* = −0.13, *p* = 0.26), race (*r* = 0.02, *p* = 0.87)], and predictive validities [competitive employment (*r* = 0.31, *p* = 0.03)] received initial support via study correlations, while convergent [e.g., intrinsic motivation (*r* = 0.32, *p* = 0.007), job interview anxiety (*r* = −0.19, *p* = 0.08)] and criterion [e.g., prior employment (*r* = 0.22, *p* = 0.046), current employment (*r* = 0.21, *p* = 0.054)] validities were limited.

**Conclusion:**

The psychometric properties of the 11-item A-MIRS ranged from strong-to-acceptable, indicating it may have utility as a reliable and valid method for assessing the job interview skills of autistic transition-age youth.

## Introduction

Currently, an estimated one million autistic transition-age youth (16–26 years old) live in the United States (1). Transition-age refers to a developmental stage characterized by youth transitioning from adolescence to adulthood and navigating the challenges and opportunities during this transitional period (e.g., completing education, developing independent living skills, pursuing employment) (2). Notably, many of these transition-age youth are accessing services during secondary or post-secondary educational programming to help facilitate their transition to the aforementioned adult activities (2). Yet, transition-age youth with autism have significantly lower employment rates as compared to non-autistic youth and their peers with other disabilities (2, 3). One critical barrier to employment for autistic transition-age youth noted by autism community members is the challenge of successfully navigating job interviews (4–7), which is critical for obtaining competitive employment (8–10).

During job interviews, potential employers make judgments about a prospective employee’s knowledge, skills, and abilities beginning with the initial greeting (11). Thus, job interview skills may be pivotal to securing competitive employment. Along these lines, pre-employment transition services (Pre-ETS) are delivered to transition-age youth with autism via secondary and post-secondary educational programs and include vocational rehabilitation services that focus on job interview skills among other job preparation needs (e.g., work-based learning experiences, workplace readiness training) (12). Additionally, research suggests approximately 90% of employed autistic transition-age youth receiving Pre-ETS interviewed prior to receiving their jobs (13). Despite the high prevalence of interviewing for jobs, no job interview interventions meet the gold standard criteria of being an evidence-based practice (14, 15). Meanwhile, a national repository of research-based interventions identified only one video-modeling job interview intervention as a “promising practice” based on a study of 15 youth with autism (15, 16).

In response to this gap in available job interview training, several studies have begun evaluating novel job interview training interventions among autistic youth and adults (17–22). However, these studies provided minimal data on the psychometric properties of the role playing methods used to evaluate job interviewing ability among autistic participants. Thus, we evaluated the psychometric properties of the Mock Interview Rating Scale (MIRS; a comprehensive assessment package including a scripted series of questions with a rating scale that are used in conjunction with video-recorded job interview role-plays for eight job scenarios) after it was adapted for autistic transition-age youth. Notably, the original MIRS was used to evaluate job interview skills in five lab-based randomized controlled trials (RCTs) among adults with serious mental illness (e.g., schizophrenia, bipolar disorder), adults with substance use disorders, veterans with posttraumatic stress disorder, and young adults with autism during which the assessment demonstrated sensitivity to change over time (19, 23–26). However, the psychometric properties for the MIRS were not reported in these aforementioned studies.

Recently, the psychometric properties of the original MIRS were assessed among 90 adults with serious mental illness participating in a community-based RCT. The results revealed the MIRS had strong internal consistency (*α* = 0.85), inter-rater reliability (ICC = 0.93), test–retest reliability (*r* = 0.82) and strong construct (social competence; *r* = 0.46, *p* < 0.001), convergent (e.g., processing speed; *r* = 0.36, *p* < 0.001), divergent (e.g., physical health; *r* = −0.11, *p* = 0.29), criterion (e.g., duration (months) at last full-time job; *r* = 0.30, *p* = 0.019) and predictive validity (e.g., job offers received by nine-month follow-up; *r* = 0.35, *p* = 0.026) (27, 28). Notably, the original MIRS was developed in 2012 and was adapted from an empirical review of the job interview construct (29) and an expert panel (30) into nine items that were scored (upon reviewing a job interview role-play) via a five-point Likert-type scale using an anchoring system (23). Specifically, the MIRS assessed one’s comfort level during the interview, negotiating time off, conveying oneself as a hard worker, sharing things in a positive way, sounding honest, sounding interested in the position, sounding easy to work with, sounding professional, and overall rapport with the hiring manager. The initial RCTs using the MIRS had difficulty capturing skills related to ‘negotiating time off’ (as participants commonly forgot to ask for time off during the interview), which led to the removal of this item in subsequent studies (27, 31).

In the present study, we evaluated the MIRS’ structure, reliability, and validity after adapting the items for transition-age youth with autism (i.e., A-MIRS) who participated in one of two RCTs. The first RCT studied whether *Virtual Interview Training for Transition Age Youth* (VIT-TAY; a job interview simulator with automated feedback systems that was designed to support autistic transition-age youth) delivered in school-based pre-employment transition services (Pre-ETS) was effective at improving employment outcomes among autistic transition-age youth (32). The second RCT evaluated whether *Virtual Reality Job Interview Training* (VR-JIT) delivered for autistic transition-age youth in high school (33). VR-JIT is a job interview simulator (delivered via the internet) with automated feedback that was originally designed for adults with mental health challenges. Notably, the autism community reviewed VR-JIT and provided feedback to adapt it into VIT-TAY (34; see methods). Both interventions are licensed commercially by SIMmersion LLC.^1^

### Aims and hypotheses

In the current study, we aimed to use standardized measurement development methods that recommended conducting exploratory and confirmatory factor analyzes along with Rasch model analytic and calibration techniques (28, 34). We assessed A-MIRS reliability via analyzes of internal consistency, inter-rater reliability, and test–retest reliability. We assessed the validity of the A-MIRS via correlational analyzes with variables representing construct, convergent, divergent, criterion and predictive validity. Construct validity (i.e., whether interview skills are accurately being measured) was assessed via the relationships between the A-MIRS and a job interview skills self-report and pragmatic social skills (35). Based on job interviewing and acquisition theoretical frameworks (29, 36–39), we hypothesized that job interview anxiety, social challenges, cognitive ability, internalizing behavior, mood, and interview training enjoyment could reasonably represent convergent validity (i.e., similar or related concepts are correlated) markers of job interviewing skill. For example, internalizing behaviors (e.g., generalized anxiety) could disrupt one’s ability to answer interview questions (40).

Regarding divergent validity (i.e., unrelated concepts are not correlated), we hypothesized that sex, age, race, and externalizing behavior would not be related with interview skills. Specifically, sex and race have not been related to work-based social skills in the autism literature (41) and the general job interview literature (29, 42). Additionally, age and externalizing behavior have not been correlated with performance-based interview skills in prior studies with autistic young adults (19, 32). Notably, externalizing behavior (e.g., rule-breaking) could be masked, and thus, not affect one’s job interview performance (43). For criterion validity (i.e., extent to which a construct correlates with real-world representation of that construct), we hypothesized that more extensive employment history (e.g., prior employment; prior job, internship, or volunteer position; learning skills during internships or volunteer work) would be associated with stronger interview skills as participants may have developed employable skills that could be discussed during the job interview (29). For predictive validity (i.e., ability to predict a future, related outcome), we hypothesized that stronger interview skills measured by the A-MIRS post-test scores would be related to subsequently obtaining competitive employment.

## Methods

### Participants

Eighty-five autistic transition-age youth (ages 16–26) were enrolled from six schools (one in Ohio, one in New Jersey, and four in Michigan) and represented suburban, urban, and rural communities as well as public, private, and charter schools. Our study participants varied in terms of their preference for identity-first (i.e., autistic) or person-first language (i.e., transition-age youth with autism). Thus, we use both identities in this dissemination of the study results. The first author led the first RCT (32), and mentored the third author who led the second RCT (33). The second RCT’s research team used methods and trainings from the first RCT to maintain fidelity of the methods and data collected.

A clinical or educational classification of autism was determined as part of the two RCTs. Participants met criteria for autism with either (a) a cutoff of a T score of 60 via teacher or parental report of the Social Responsiveness Scale, Second Edition (SRS-2) (44); or (b) a diagnosis of autism recorded in the student’s individualized education program that used disability classification via the Individuals with Disabilities Education Act (2014). Additionally, all participants are: (a) at a 3rd grade reading level (or higher) via the sentence comprehension subtest of the Wide Range Achievement Test, Fourth Edition (45), (b) currently receiving transition services or Pre-ETS, (c) willingness to be recorded on video, and (d) providing informed consent/assent. Participants were excluded if they had: (a) an uncorrected visual or hearing impairment that would prevent the student from using the interventions, or (b) a medical illness that compromised their cognitive ability to engage with the intervention. The Institutional Review Boards at the University of Michigan (HUM00129575) and the Kessler Foundation (R-1036-18) approved the studies. All participants aged 18 and older were independent and provided informed consent without a conservator. All participants under the age of 18 provided written parental consent and their own written assent.

### Recruitment

The research teams led community-based presentations at local educational conferences and meetings as well as cold-calls (or emails) to local public, private, and charter schools. Once schools expressed interest in the study, we presented findings from prior studies and discussed the design of the RCT and intervention implementation with school administration and staff. Once schools committed to a partnership, the research team and schools worked together to recruit student participants. Recruitment methods included hosting informational meetings for families, and school partners sending general study information to families (e.g., contact information for study team members). Participants or their families then reached out to the study team for more information and to begin enrollment.

### Study procedures

A Data and Safety Monitoring Board reviewed, approved, and monitored study procedures for both trials. Study measures were collected or administered by research staff. The research team was trained by the PI or project manager, and monitored for data collection fidelity. All participants completed baseline data collection over two study visits prior to being randomly assigned to the Pre-ETS/transition services with virtual interviewing group or the Pre-ETS/transition services only group. Details on the order of assessments can be found here (32). Notably, we evaluated the baseline data across all participants in both RCTs. However, we only evaluated predictive validity using the sample from the first RCT as the second RCT did not collect follow-up data on employment outcomes.

### Measures

#### Background characteristics and baseline vocational history

Teachers completed surveys on all participants’ background characteristics that included age (computed using birthdate and date of consent), sex assigned at birth, grade level (0 = freshman, sophomore, junior, or senior in high school, 1 = transition year), co-occurring disability (via education record: autism, intellectual disability, emotional disturbance, other health impairment, specific learning disability [46]), and parental educational attainment (highest obtained by mother or father). Participants completed surveys about their vocational history (e.g., prior and current employment status, internships, and volunteer work) during their baseline visit. Vocational history data not provided by participants were obtained via parents or teachers via educational records.

#### Job interview skills (performance-based)

Participants completed a single, video-recorded job interview role-play at their baseline visit as part of the A-MIRS assessment. As noted previously, the A-MIRS is an adaptation of the original MIRS. Specifically, the MIRS is an evaluation package consisting of fifteen scripted questions (delivered by role-players) answered by participants for one of eight jobs. Role-play performances were captured via video and scored on nine skills by masked raters: 1) comfort level, 2) negotiation, 3) hard worker, 4) sounding easy to work with, 5) sharing things in a positive way, 6) sounding interested, 7) sounding professional, 8) sounding honest, and 9) overall rapport. Raters used an anchoring system with a Likert-type, five-point scale. The identified skills were derived from community partner feedback and the job interview literature (30, 47) and were originally built into the foundation of VR-JIT (23). Notably, the “overall rapport” domain was unique to the MIRS and not emphasized as a skill via the VR-JIT feedback system.

To adapt the MIRS for use with transition-age youth with autism, we followed a similar approach as the original MIRS where skills were initially identified for an intervention. First, we used a community-engaged approach [detailed here Smith et al. (48)] where we enrolled 45 autism community members (i.e., *n* = 24 autistic transition-age youth, *n* = 4 employed autistic adults, and *n* = 17 parents, teachers, community employers) to review the VR-JIT intervention (and eight job interview skills targeted by the intervention) and then made recommendations to tailor the skills to serve autistic transition age youth. These recommendation were processed, summarized, and validated using member checking (49, 50). We then shared the results with a community advisory board (consisting of an autistic transition-age youth, a former president of the Michigan State Board of Education, a transition manager for a local school district, a local transition teacher, a local business owner, three local clinical and educational service providers, and two administrative representatives from a national advocacy group). The community advisory board validated the member feedback and provided their own recommended adaptations to VR-JIT. Finally, a scientific advisory board validated prior recommendations and provided their own suggested adaptations (48). Second, the autism community recommendations were implemented for the youth version of VR-JIT called *Virtual Interview Training for Transition-Age Youth* (VIT-TAY). Specifically, the autism community partners recommended removing the negotiation skill and renaming seven skills: 1) “comfort level” became “being confident”; 2) “sounding professional” became “being professional”; 3) “sharing things in a positive way” became “being positive”; 4) “sounding interested” became “showing interest”; 5) “sounding honest” became “being honest”; 6) “hard worker” became “being dependable or hard working”; and 7) “sounding easy to work with” became “working well with others”). Then three new skills were added to VIT-TAY: 1) “sharing strengths,” 2) “sharing past experiences,” and 3) “sharing limitations.”

Third, a similar pattern emerged when transitioning from the item names on the MIRS to the item names on the A-MIRS: 1) the negotiation item was removed, 2) “overall rapport” item was retained, and 3) the remaining seven MIRS items were renamed for the A-MIRS (to be consistent with the skills targeted in VIT-TAY). Specifically, the A-MIRS includes the following 11 items: 1) confidence (formerly “comfort” in the MIRS), 2) being positive (formerly “sharing things in a positive way” in the MIRS), 3) professionalism (formerly “sounding professional” in the MIRS), 4) showing interest (formerly “sounding interested” in the MIRS), 5) honesty (formerly “sounding honest” in the MIRS), 6) being dependable or hard working (formerly “hardworker” in the MIRS), 7) working well with others (formerly “sounding easy to work with” in the MIRS), 8) sharing strengths and skills (not included in the MIRS), 9) sharing past experiences (not included in the MIRS), 10) sharing past limitations (not included in the MIRS), and 11) overall rapport.

Fourth, the original MIRS used a five-point scale (23) that was adapted to use a seven-point scale on the A-MIRS in order to capture greater variation in interviewees’ skill and more precision in raters’ scoring. The fifth aspect of adapting the MIRS into the A-MIRS was with respect to revising our use of the anchors to accommodate the new seven-point Likert-type scale. Thus, to simplify as well as strengthen the use of the anchors, our team used their expertise in autism and interview behavior to ensure the anchors for each item matched the construct for which it was intended. The anchors were also written based on the interview script which was heavily guided by needs of the autistic population (no idioms, etc.). The final adaptation was to change the job scenarios available in the MIRS to reflect the same jobs portrayed in VIT-TAY.

Thus, to complete the A-MIRS, participants reviewed 14 scenarios (Appendix A) for part-time jobs (e.g., tech support, web developer, stock clerk, cashier, food services). Participants then had approximately 5 minutes to prepare for their interview role-play. The A-MIRS role-players were research assistants trained to perform the role of a friendly hiring manager. The A-MIRS included 15 required job interview questions to be asked by the role-player along with up to 10 additional questions selected at random (see Appendix B). The research assistants were trained using the same methodology implemented in a series of prior studies [e.g., (19, 24)]. Fidelity of the role-plays were evaluated based on asking all 15 required questions using a checklist that role-players completed during the mock interview. The role-plays lasted approximately 15 min each and were video-recorded.

The recorded mock interview videos were randomly assigned to three raters in RCT 1 and two raters in RCT 2 who were masked to condition and had experience conducting real-world job interviews. Raters trained to a scoring standard using four gold standard practice videos prior to independently rating the study videos. The A-MIRS total score was computed by summing 11 items (ranging from 1 to 7 points per item) for each video performance. The A-MIRS scoring scheme (including anchors) can be found in Appendix C. Additionally, we implemented random double coding for approximately 25% of the videos. This approach aimed to prevent coding drift and involved the coding trainer meeting with the coders to collectively examine and address any discrepancies in coding. Specifically, they focused on inconsistencies where the assigned codes differed by more than one point within a specific domain. Through collaborative discussions, the goal was to arrive at a consensus score that reflected a shared understanding among the coders.

#### Job interview skills (self-report)

Each participant completed the 10-item self-report *Measure of Job Interview Skills* (MOJO-iSkills), which recently demonstrated initial reliability and validity in a recent study of transition age youth with autism (51). The items were assessed via 5-point Likert-type ratings (1 = not at all true to 5 = very true) and we used a scaled T score in our analyzes. The survey was completed by participants after the job interview role-play. Internal consistency was strong (*α* = 0.94).

#### Pragmatic social competence

We assessed 12 domains of pragmatic social competence (i.e., fluency, clarity, focus, intonation, body language, facial expressions, eye contact, social appropriateness, reading social cues, connection, perspective-taking, and overall conversation). Two independent raters (who did not code the aforementioned job interview role-plays using the A-MIRS) were trained to apply an existing scoring rubric to the job interview role-play videos captured at baseline. Specifically, the raters used the scoring anchors from the *Social Skills Performance Assessment for Autism Spectrum Disorders and Related Conditions* (52, 53). The scale had excellent internal consistency (*α* = 0.97) and excellent inter-rater reliability (ICC = 0.96 at pre-test; ICC = 0.94 at post-test).

#### Job interview anxiety (self-report)

Each participant completed the 11-item self-report *Measure of Job Interview Anxiety* (MOJO-iAnxiety), which recently demonstrated initial reliability and validity in a recent study of autistic transition age youth (51). The items were assessed via a 3-point Likert-type ratings (1 = not at all to 2 = often) and we used a scaled T score in our analyzes. The survey was completed by participants after the job interview role-play. Internal consistency was strong (*α* = 0.85).

#### Job interview intrinsic motivation

We surveyed participants regarding their intrinsic motivation to prepare for job interviews. We adapted the 7-item interest/enjoyment subscale of the Intrinsic Motivation Inventory (54). First, we reviewed the subscale for accessibility at a 4th grade reading level and item redundancy. The subscale included three items that asked about “enjoyment” of job interview practice in three slightly different ways so we removed two of these items to eliminate redundancy. Second, the original IMI scaled the items on a 7-point Likert-type scale from 1 = not at all true to 7 = very true. To increase accessibility and scale comprehension, we rescaled the measure to a 5-point Likert-type scale from 1 = not at all true to 5 = very true. The internal consistency of this five item adapted scale was strong *α* = 0.84.

#### Social challenges

We obtained data on social challenges through parent or teacher-reports using the SRS-2 (44) during the study inclusion visit. The SRS-2 is a 65-item assessment of autistic traits as observed by a rater (e.g., parent, teacher). The SRS-2 generates ratings for one’s social communication, social cognition, social awareness, restricted interests and repetitive behaviors, and social motivation. Ratings are scaled from 0 = never true to 3 = almost always true. The assessment generates an overall T-score that we used in our analyzes. Internal consistency was strong (*α* = 0.97).

#### Cognitive ability

Participants were assessed using the National Institutes of Health (NIH) Toolbox Cognition Battery. This assessment took place on a different day than the A-MIRS job interview role-plays. The battery consisted of seven computerized tests that required approximately 1 h to complete. The toolbox generated composite scores based on its seven tests including a Crystallized Cognition Composite [i.e., Picture Vocabulary Test, Oral Reading Recognition Test (internal consistency was acceptable; α = 0.71)] and a Fluid Cognition Composite [i.e., Dimensional Change Card Sort Test; the Flanker Inhibitory Control and Attention Test, the Picture Sequence Memory Test, the List Sorting Working Memory Test, and the Pattern Comparison Processing Speed Test (internal consistency was acceptable; *α* = 0.70)] (55, 56). Fully corrected T-scores for crystallized cognition (i.e., knowledge and skills) and fluid cognition (i.e., use of logic and problem solving) were used in our analysis.

#### Depressive symptoms

The shortened version of the *Mood and Feelings Questionnaire* [MFQ; Angold et al. (57)] was used to assess depressive symptoms. The MFQ has 13 items that focus on one’s feelings and behavior over the past 2 weeks. Participants responded on a scale of 0 = not true, 1 = somewhat true, and 2 = true. The MFQ has been evaluated among youth with autism and was found to be sensitive to depression (58). Internal consistency was strong (*α* = 0.83).

#### Behavioral challenges

Parent- or teacher-reports via the Achenbach standardized *Child Behavior Checklist* (CBCL) or *Adult Behavior Checklist* (ABCL) (59, 60). The CBCL/ABCL rated 118 trait behaviors that assess both internalizing [i.e., anxious/depressed, somatic complaints, withdrawn/depressed (CBCL only)] and externalizing behaviors [i.e., rule-breaking, intrusive, and aggressive behavior (ABCL only)]. The behaviors are rated on a three-point scale of 0 = not true, 1 = somewhat true, and 2 = very true. The assessment generates T-scores that we used in our analyzes. Internal consistency was acceptable (*α* = 0.78).

#### Employment outcomes

At six-month follow-up, we collected data (from participants or from parents or teachers if participants were unavailable) on whether the participants competitively obtained a job or secured employment through informal means (i.e., participants obtained their position after completing an internship or volunteer position). Thus, employment outcomes were coded as competitive employment (1 = yes, 0 = no), informally obtained employment after completing an internship or volunteer position (1 = yes, 0 = no).

### Missing data

Missing data were imputed via the SPSS expectation–maximization algorithm missing values analysis package (61). This maximum likelihood estimation method generates unbiased estimates that provide less biased parameter estimates as compared to regression or mean imputation (62). For Trial 1, total scores were imputed for participants on the following measures: 1) crystallized cognition (*n* = 2), 2) fluid cognition (*n* = 3), 3) SRS-2 (*n* = 1), 4) MFQ (*n* = 1), and 5) CBCL/ABCL (*n* = 3). In addition, item-level data were imputed for participants on the following measures: 1) MOJO-iSkills (*n* = 1), 2) job interview intrinsic motivation (*n* = 1), 3) MOJO-iAnxiety (*n* = 2), and 4) MFQ (*n* = 3). Analyzes were conducted with and without the imputed data. Given that the magnitude and direction of the effects did not differ, we used the full sample with imputed data.

### Analyzes of participant characteristics

We used descriptive statistics (mean, standard deviation, percentage) to report on participant demographics, employment history, clinical and cognitive characteristics (used in the validity analyzes).

#### Psychometric analyzes

We evaluated the data for normality and no transformations were needed. We characterized the sample using raw frequencies, means, and standard deviations. We developed our measure employing published measurement development standards (63). Our process included analyzes associated with: item and measure initial screening; dimensionality assessment; item misfit, bias, and calibration; A-MIRS scores and scoring; and score reliability and validity. State-of-the-science psychometrics were used in our measure development and aimed to obtain a unidimensional item set. We conducted analyzes in a sequential and sometimes iterative fashion, independently conducting each analysis, yet informing subsequent analyzes as important findings were identified.

##### Exploratory factor analysis (EFA)

We employed Exploratory Factor Analysis (EFA) without any pre-existing theories to identify a comprehensive set of items. In order to determine essential unidimensionality, we considered it supported if the ratio of eigenvalue 1 to eigenvalue 2 was equal to or greater than 4.0, and if eigenvalue 1 accounted for at least 40% (0.40) of the total variance (34, 64–67). Mplus software version 7.4 was utilized to conduct the EFA (version 7.4; 68). Within the framework of Classical Test Theory, we assessed whether to exclude items based on sparse cell frequencies (response categories with fewer than 10 respondents across items), low Pearson r item-rest score correlations (less than 0.40), or non-monotonicity. To examine monotonicity, we employed a non-parametric model to create and evaluate item-rest plots and an expected score-by-latent trait plot (Testgraf Software; 69).

##### Confirmatory factor analysis (CFA)

We utilized a unidimensional, single-factor Confirmatory Factor Analysis (CFA) to validate the unidimensionality of the item set (70–72). Items with factor loadings below 0.50 or exhibiting local dependence (residual correlation exceeding 0.20; correlated error modification index equal to or greater than 100) were considered for potential exclusion (70–72). Unidimensionality was deemed supported when the overall model fit criteria met the following thresholds: a comparative fit index (CFI) equal to or greater than 0.95, a Tucker-Lewis index (TLI) equal to or greater than 0.95, a root mean square error of approximation (RMSEA) less than 0.10, and a standardized root mean residual (SRMR) less than 0.08 (64, 65, 73, 74). If the overall fit criteria were not fully met, we employed Confirmatory Bifactor Analysis (CBFA) to evaluate whether the multidimensional data were sufficiently “unidimensional” to fit a unidimensional measurement model (72, 75). In terms of assessing factor strength, an omega-H index value derived from CBFA exceeding 0.8 has been suggested as a threshold for unidimensionality (76). Both CFA and CBFA were conducted using Mplus [version 7.4; Muthén et al. (68)].

##### Rasch analysis

The item parameters for a unidimensional item set were estimated using the constrained (common threshold) Andrich rating scale model (RSM) (77). This specific version of the Rasch model, which utilizes a common threshold, is suitable when the standard sample size requirements for the Rasch partial credit model are met, specifically if the sample size (N) is equal to or greater than 50 and if response categories have at least 10 respondents within each item. Items that exhibited significant misfit to the RSM, as indicated by a standardized chi-square to degrees of freedom (S-X2/df) effect size greater than 3, were eliminated (74).

##### Differential item functioning (DIF)

We examined Differential Item Functioning (DIF) to identify any potential biases towards or against specific subgroups by assessing item bias. Using Andrich rating scale model (RSM) item parameter estimation, we conducted exploratory DIF analyzes with approximately 30 participants per DIF factor subgroup (78–80). The DIF factors we investigated included age in years (<18.5 vs. ≥18.5), race/ethnicity (White vs. Other), education (high school freshman to senior vs. adult transitional), and co-occurring disability (autism only vs. autism plus other disabilities). Items were considered for removal if they displayed significant DIF, determined by two criteria: (a) a group-specific difference in item parameters with a value of p of ≤0.05 and a DIF contrast effect size of ≥0.64 for each tested item, and (b) more than 2% of DIF-corrected vs. uncorrected score differences exceeded the standard errors for individual uncorrected scores (81). Rasch and Rasch-based DIF analyzes were conducted using Winsteps software [version 3.1.2; Cai et al. (82)].

A final Confirmatory Factor Analysis (CFA) was conducted on the retained item set of the measure to confirm its essential unidimensionality status. The same overall model fit criteria, as described earlier, were applied. Once the final set of items for the A-MIRS was identified, the RSM was employed to determine item parameters, and a T-score metric was established, centered on individuals, with a mean of 50 and a standard deviation of 10. This approach facilitated individual scoring by calibrating item measurements and enabled subsequent assessments of reliability and validity.

#### A-MIRS distribution characteristics

We generated the minimum and maximum observed scores, mean, standard deviation, median, skewness, excess kurtosis, and the percentage of participants with the minimum or maximum possible score (indicating potential floor or ceiling effects). It is worth noting that we considered floor and ceiling effects as acceptable if they affected ≤20% of respondents with minimum or maximum scores (83, 84). Moreover, skewness and kurtosis values within the range of −1.0 to +1.0 were considered indicative of essential normality (85). To facilitate scoring without relying on a Rasch RSM anchored-parameter computer program, we created a table (provided in Appendix D) that allows for the conversion of raw summed scores to T scores, providing an accessible alternative.

#### Inter-rater reliability

The PI provided training to three role-play raters by utilizing four mock job interview videos considered as gold standards. The raters independently scored all the videos, and subsequent discussions took place between the PI and the raters to reach a consensus on the gold standard rating for each of the four videos. Throughout the study, the raters were expected to score around 10% of all the videos, which would then be assessed for inter-rater reliability using intraclass correlation coefficient analysis.

#### Internal consistency

We computed internal consistency reliability using Cronbach’s alpha and overall reliability using Rasch/IRT-based methods, where reliability was determined by the formula 1 – (median SE2 / SD2) (86). We defined reliability as “excellent,” “good,” or “acceptable” based on specific criteria: excellent if reliability was ≥0.90, good if reliability was ≥0.80 but less than 0.90, and acceptable if reliability was ≥0.70 but less than 0.80. Reliabilities of ≥0.70 were considered suitable for group-level comparisons, while reliabilities of ≥0.90 were considered appropriate for individual-level comparisons (87). We identified score ranges that had score-level-specific reliabilities of ≥0.70 or higher, indicating that they were acceptable or better in terms of reliability.

#### Test–retest reliability

We assessed test-retest reliability via the correlation between the A-MIRS pre-test variable and the A-MIRS post-test variable (using only RCT participants randomized to Pre-ETS only). This approach will eliminate any potential bias introduced in the VIT-TAY group due to their use of the intervention targeting job interview skills.

#### Validity analyzes

We conducted point serial and Pearson correlations to test the relationships between the A-MIRS T-score and the variables representing measurements of validity. For construct validity, we correlated the A-MIRS T-score with self-reported interview skill and pragmatic skill. For convergent validity, we correlated the A-MIRS T-score with self-reported job interview anxiety, intrinsic motivation to practice interviewing, grade level, social challenges, cognitive ability, internalizing behavior, and depressive symptoms. For divergent validity, we correlated the baseline A-MIRS T-scores with measures of age, sex, race (% Black, Indigenous, Persons of Color), and externalizing behaviors. For criterion validity, we correlated the baseline A-MIRS T-scores with prior employment; a prior job, internship, or volunteer position; learning skills during internships or volunteer work. For predictive validity, we correlated the post-test A-MIRS T-scores with obtaining competitive employment and non-competitive employment by 6-month follow-up.

Given the Pre-ETS only group did not obtain competitive employment by 6 months and the potential bias of receiving VIT-TAY, we independently report predictive validity correlations in both study groups. Notably, the 2nd RCT did not collect follow-up employment outcome data and were not included in the predictive validity analyzes. In our validity analyzes, statistically significant correlation magnitudes of >0.3 in absolute value were required (88, 89). Notably, we accounted for factors that could potentially influence the strength of validity coefficients, including skewness, scale and criterion scale reliability, timing, range restriction, and method variance. It is worth highlighting the presence of method variance, which indicates that self-report evaluations and external rater assessments (such as role-play ratings) will exhibit weaker correlations compared to assessments made using a consistent method (90). Thus, moderately-sized correlations still provide meaningful information regarding validity (predictive, criterion, convergent, and construct) (91).

#### Sample size requirements

To conduct CFAs, it is recommended to have a minimum of five cases (*n* = 5) per observed variable, particularly when modeling a single underlying variable with multiple indicators (92). Therefore, a sample size of *n* = 85 would be sufficient. For RSM-based analyzes, current guidelines suggest that a minimum of minimum *N* = 50 participants is necessary to establish stable item parameters, considering that response categories have *n* ≥ 10 respondents across items (93). Furthermore, when performing exploratory DIF analyzes in conjunction with RSM estimation, it is advised to have approximately 30 participants per subgroup (78).

## Results

### Participant characteristics

Participants were M = 19.3 (SD = 2.8) years old, predominately male (83.5%) and the majority were in White (60%) and in their transitional year of education (44.7%; remaining participants were seniors, juniors or sophomores). Also, 42.4% of participants had at least one additional co-occurring disability (e.g., specific learning disability, other health impairment). Regarding parental socioeconomic status, 64.6% of participants had at least 1 parent who completed an undergraduate or graduate degree. Additional details on participant demographics and employment history can be found in Table 1. Descriptive statistics (mean, standard deviation, percentage) regarding variables evaluated as validity factors are reported in Table 2.

**Table 1:** Participant background and baseline work history characteristics (*n* = 85).

	Mean (SD) or %
Age	19.3 (2.8)
Sex assigned-at-birth (% male)	83.5%
Race	
White	60.0%
Black or African American	22.4%
Latinx	8.2%
More than one race	5.9%
Asian American	2.4%
Indigenous American	1.2%
Education level (% in transition year)	44.7%
Any co-occurring disability	42.4%
Specific learning disability	10.6%
Emotional disturbance	9.4%
Intellectual disability	12.9%
Other health impairment	17.6%
Highest parental education completed (% undergraduate or graduate degrees)	64.6%
Baseline employment history	
Ever had a job in the community	34.1%
Currently have a job	8.2%
Ever had a job, internship, or volunteer position	78.8%
Currently have a job, internship, or volunteer position	43.5%
Did you learn skills in this internship or volunteer position for a future job (*n* = 62)	91.9%

Pre-ETS, Pre-employment transition services; VIT-TAY, Virtual Interview Training for Transition-Age Youth.

**Table 2:** Social, cognitive, behavioral, and employment follow-up variables (*n* = 85).

	Mean (SD) or %	Range
Job interview skills (self-reported)	50.0 (10.1)	30.0–66.5
Pragmatic social skills (item level; *n* = 71)	3.1 (0.7)	1.7–4.3
Job interview anxiety	50.0 (10.1)	26.6–72.5
Intrinsic motivation for practicing job interviews	18.6 (4.8)	8.0–25.0
Social challenges (*n* = 81)	65.9 (13.1)	43.0–96.0
Crystallized cognition (*n* = 69)	41.4 (11.6)	26.0–82.0
Fluid cognition (*n* = 66)	30.5 (11.9)	9.0–65.0
Internalizing behavior (adult behavior checklist; *n* = 69)	57.7 (10.4)	31.0–76.0
Externalizing behavior (adult behavior checklist; *n* = 69)	51.9 (10.6)	32.0–78.0
Depressive symptoms	6.0 (4.5)	0.0–21.0
Employment outcomes (six-month follow-up; *n* = 71)		
Pre-ETS only (*n* = 23)		
Non-competitive employment (% yes)	30.4%	–
Obtained competitive employment (% yes)	–	–
Pre-ETS + VIT-TAY (*n* = 48)		
Non-competitive employment (% yes)	16.7%	–
Obtained competitive employment (% yes)	25.0%	–

### Psychometric analyzes

Our EFA analysis strongly supported attaining an essentially unidimensional measure for A-MIRS. This was indicated by a favorable eigenvalue 1-to-2 ratio of 5.4, with eigenvalue 1 accounting for 54.7% of the modeled variance, while eigenvalue 2 accounted for 10.2% of the variance. Regarding classical test theory, none of the original 11 A-MIRS items were excluded based on criteria such as low item-rest correlations, sparse response option cells, or non-monotonicity.

During the CFA, none of the 11 items were eliminated based on low factor loadings, and there were no exclusions due to high residual correlations or high correlated error modification index values. The unidimensional model demonstrated good-to-excellent overall fit, with the observed fit index values meeting the recommended criteria, except for the RMSEA value (i.e., CFI = 0.97, TLI = 0.96, RMSEA = 0.10, and SRMR = 0.07). Because our CFA model RMSEA value was slightly above targeted fit threshold, we conducted a CBFA, which strongly supported the unidimensionality of the 11-item A-MIRS measure (i.e., omega-H = 0.85). Consequently, the results of the CBFA demonstrated that a single, overarching factor was primarily accountable for the consistent variation in scores.

In the initial Rasch modeling of the 11 items, none of the items were eliminated based on item misfit, and no items were excluded due to DIF associated with the investigated potentially biasing factors (age, race, education, and co-occurring disabilities). The final CFA model remained unchanged from the original 11-item model and demonstrated good-to-excellent overall fit. The CBFA results further confirmed the essential unidimensionality of the model. Therefore, the A-MIRS measure contains 11 items. Using the RSM, we calculated the item thresholds (b values) for the measure, which spanned from 3.5 to 87.0 in the T score metric. Measure content is presented in Table 3.

**Table 3:** Items for autism mock interview rating scale.

1. Being confident 2. Being positive 3. Being professional 4. Showing interest in the position 5. Being honest 6. Being dependable or hardworking 7. Working well with others 8. Sharing strengths and skills 9. Sharing past experiences 10. Sharing past limitations 11. Overall rapport No items eliminated

#### A-MIRS distribution characteristics

**Figure 1:**
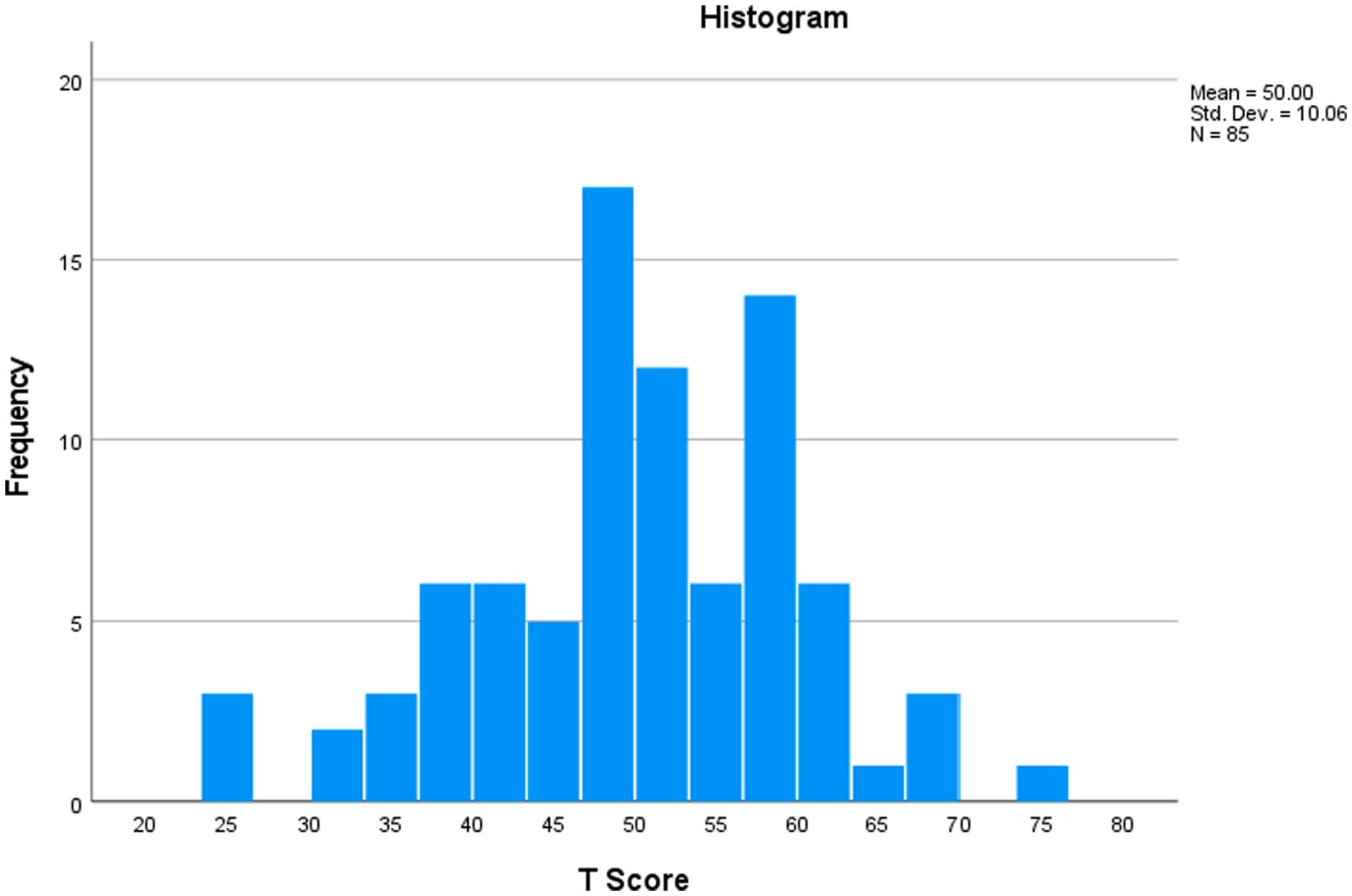
A-MIRS T score distribution.

The A-MIRS score distribution characteristics are presented in Table 4. The score distribution was found to be essentially normal, as indicated by skewness and excess kurtosis values of −0.31 and 0.43, respectively. There were no floor or ceiling effects observed, as none of the respondents achieved the minimum or maximum possible scores, resulting in a percentage of 0% for both cases. A histogram of the measure’s score distribution is presented in Figure 1. Observed T scores ranged from 23.6 to 76.2. Note that possible T scores range from −16.3 to 110.2 (see the conversion or “lookup” table with associated T score SEs, i.e., Appendix D). The lookup table presents a convenient and user-friendly alternative when compared to Rasch RSM anchored-parameter scoring.

**Table 4:** T score distributions.

	A-MIRS T score
*N*	85
Mean	50.0
Median	50.7
SD	10.1
Skewness	−0.3
Kurtosis	0.4
Minimum observed	23.6
Maximum observed	76.2

A-MIRS, Autism Mock Interview Rating Scale.

#### Reliability

##### Interrater reliability

In the first RCT, three raters coded all 71 pre-test and 70 post-test videos. To establish interrater reliability, the three raters coded the same seven pre-test videos (ICC = 0.97). Due to raters leaving the project, Rater 1 and rater 2 scored six post-test videos (ICC = 0.94), Rater 1 and rater 3 scored seven post-test videos (ICC = 0.97), and Rater 2 and 3 scored 8 post-test videos (ICC = 0.97). In the second RCT, two raters scored all 14 pre-test and 14 post-test videos and established reliability by coding four videos (ICC = 0.95). To prevent drift, 25% of the videos were randomly selected to be double coded and the first author met with the coding team to address and resolve any coding inconsistencies, specifically focusing on cases where there was a discrepancy of more than one point on a particular item. The meeting contained discussion to reach a consensus on the final score for such cases.

##### Internal consistency

Cronbach’s alpha coefficient demonstrated excellent reliability (0.90), and the SE-based reliability was also excellent (0.91). Concerning specific score levels, the reliabilities of A-MIRS T scores ranging from 0 to 90 were acceptable or better (≥0.70), while T scores between 28 and 62 exhibited excellent reliabilities (≥0.90).

##### Test–retest reliability

Among the Pre-ETS only group from the parent RCT, the 11-item A-MIRS T score at pre-test was correlated (*r* = 0.84, *p* < 0.001) with the 11-item A-MIRS T score at post-test (*n* = 22; one participant failed to complete their post-test role play).

#### Validity

##### Construct and convergent validities

Construct validity of the A-MIRS was established through significant correlations with the following measures, meeting the correlation threshold of ≥0.30 (refer to Table 5): self-reported job interview skills (*r* = 0.34, *p* = 0.001) and pragmatic social skills (*r* = 0.61, *p* < 0.001). Meanwhile, intrinsic motivation to practice job interviewing was correlated with the A-MIRS above the required threshold (*r* = 0.32, *p* = 0.007) in support of convergent validity. Although crystallized cognition was significantly related with the A-MIRS (*r* = 0.26, *p* = 0.03), it did not met the magnitude threshold. Notably, the job interview anxiety was related to the A-MIRS in the expected direction but did not meet the magnitude threshold or obtain significance (*r* = −0.19, *p* = 0.08). Grade level, social challenges, fluid cognition, internalizing behavior, and depressive symptoms were not related with the A-MIRS (all *p* > 0.10).

**Table 5:** Validity correlations (*n* = 85).

	A-MIRS	
	r	*p*
Construct validity		
Job interview skills (self-reported)	0.341	0.001
Pragmatic social skills (*n* = 71)	0.605	<0.001
Convergent validity		
Job interview anxiety	−0.189	0.084
Intrinsic motivation to practice interviewing	0.318	0.007
Grade level (adult transition vs. senior or lower)	−0.176	0.107
Social challenges	−0.010	0.931
Crystallized cognition	0.263	0.029
Fluid cognition	0.022	0.858
Internalizing behavior	0.157	0.198
Depressive symptoms	−0.042	0.699
Divergent validity		
Age	−0.125	0.256
Sex	0.119	0.277
Race (% Black, Indigenous, and other People of Color)	0.018	0.868
Externalizing behavior	0.182	0.133
Criterion validity		
Ever had a job in the community (% yes)	0.217	0.046
Currently have a job (% yes)	0.210	0.054
Ever had a job, internship, or volunteer position (% yes)	0.260	0.016
Currently have a job, internship, or volunteer position (% yes)	0.188	0.084
Learned skills in internship or volunteer position for future (% yes; *n* = 59)	0.232	0.077
Predictive validity (via 6-month follow-up)		
Pre-ETS only (*n* = 23)		
Non-competitive employment (% yes)	−0.098	0.656
Obtained competitive employment (% yes)	–	–
Pre-ETS + VIT-TAY (*n* = 48)		
Non-competitive employment (% yes)	−0.146	0.328
Obtained competitive employment (% yes)	0.312	0.033

##### Divergent, criterion, and predictive validities

Divergent validity of the A-MIRS was supported as the following measures did not exhibit significant correlations and failed to meet the required correlation magnitude threshold: age, sex, race, and externalizing behavior (all *p* > 0.10; see Table 5). For criterion validity, two variables were significantly related to the A-MIRS in the expected direction (i.e., ever had a job [r = 0.22, *p* = 0.046] and ever had a job, internship or volunteer position [r = 0.26, *p* = 0.02]), but did not reach the required 0.30 threshold. Current employment; current job, internships, or volunteer position; and learned skills in these positions for future jobs did not meet the 0.30 threshold though trended towards significant (all *p* < 0.10). For predictive validity (Table 5), obtaining competitive employment in the Pre-ETS + VIT-TAY group was significantly correlated with the A-MIRS (*r* = 0.31, *p* = 0.03) and met the 0.30 threshold. Meanwhile, obtaining non-competitive employment was not significantly correlated with the A-MIRS for either group (Pre-ETS only and Pre-ETS + VIT-TAY; both *p* > 0.10).

## Discussion

Job interview skills are both critical targets for intervention and highly relevant for obtaining competitive employment (13). Moreover, the autism community specifically identified the need for job interview training and employer-facing interventions (4, 7). While efforts are emerging to begin focusing on employer-facing interventions, the field of vocational rehabilitation continues to support autistic people choosing to prepare for job interviews. Although research is limited in terms of how often job interview role-play training occurs, our recent evaluation of job interview training in 47 Pre-ETS programs located in Michigan, Illinois, and Florida yielded that each program provided job interview role-play training for their students with autism (94).

Given that the broad field of vocational rehabilitation has limited access to psychometrically validated job interview skills assessments and that an emerging job interview intervention literature uses role-play assessments with limited psychometric validation (18, 20–22, 32, 33), this study evaluated the structure, reliability, and validity of the A-MIRS among 85 autistic transition-age youth who participated in one of two RCTs.

Our standardized approach of EFA, CTT, CFA, CBFA, Rasch modeling, and calibration analyzes revealed an 11-item unidimensional construct for the A-MIRS. The assessment of potential Differential Item Functioning (DIF) did not identify any indications of item bias in the functioning of the A-MIRS for subgroups of transition-age youth with autism. Therefore, the A-MIRS is likely to operate in a comparable manner across autistic people from BIPOC communities (compared to White youth with autism), are high school freshmen to seniors (compared to students in transitional year), are younger than 18.5 years old (compared to older than 18.5 years), or have co-occurring disabilities (compared to those with no co-occurring disability).

The results of this study infer the A-MIRS is a reliable assessment of job interview skill among autistic transition-age youth. For instance, the internal consistency of the A-MIRS was excellent (*α* = 0.90). The obtained alpha coefficient, which surpassed the recommended threshold of 0.70 commonly used in research settings for group-level comparisons, indicated high internal consistency reliability (95, 96) and met the alpha coefficient of 0.90 real-world applied settings (96). The inter-rater reliability of the A-MIRS was strong among three raters in RCT 1 (e.g., ICC = 0.97) and two raters in RCT 2 (ICC = 0.95). The test–retest reliability findings infer that the A-MIRS is stable over time (*r* = 0.84). Additionally, the A-MIRS exhibited sensitivity to detect changes over time in two RCTs (32, 33). In these trials, the A-MIRS scores demonstrated an increase over time among groups utilizing virtual interview training compared to groups receiving services-as-usual, thereby indicating the A-MIRS effectiveness at capturing change over time.

Evidence from our validity analyzes suggests there is some initial support for the construct validity of the A-MIRS. Notably, self-reported job interview skills (*r* = 0.341, *p = 0*.001) and pragmatic social skills (*r* = 0.605, *p < 0*.001) were both significant and met the minimum *r* = 0.30 threshold (88, 89). The findings regarding the convergent validity of the A-MIRS were inconclusive, as only one variable (intrinsic motivation to practice interviewing) demonstrated a significant correlation (*r* = 0.318, *p* = 0.007) that met the required threshold of 0.30 in magnitude. Although crystallized cognition was significantly related with the A-MIRS the magnitude (*r* = 0.263, *p = *0.029) was below 0.30. Meanwhile, job interview anxiety was related to the A-MIRS in the hypothesized direction but was not significant with a magnitude below 0.30 (*r* = −0.189, *p = *0.084). Grade level, fluid cognition, internalizing behavior and depressive symptoms were not significantly related with the A-MIRS. Thus, the overall evidence for convergent validity was mixed.

As hypothesized, age, sex, race, and externalizing behavior were not correlated with the A-MIRS (29, 41, 42), which suggests the presence of divergent validity. The support for criterion validity was limited as the A-MIRS was significantly correlated (but with magnitudes below 0.30) with the variables ever having a job, internship, or volunteer position (*r* = 0.260, *p = *0.016) and ever having a job in the community (*r* = 0.217, *p = 0*.046). Additional criterion validity variables (currently have a job, currently have a job, internship, or volunteer position, learned skills in internship or volunteer position for future) were correlated with the A-MIRS at the trend level with magnitudes below 0.30. Regarding predictive validity, we evaluated the relationship between the A-MIRS and future employment in the study groups separately to remove potential bias created by using the virtual interview training. In the Pre-ETS + VIT-TAY group, the relationship between post-test A-MIRS and competitive employment within 6 months was both significant and met the 0.30 threshold (*r* = 0.312, *p = *0.033) providing initial evidence of predictive validity (88, 89). Moreover, this result is consistent with the findings from the psychometric evaluation of the original MIRS that observed a relationship between the post-test MIRS score and job offers received (28). Meanwhile, post-test A-MIRS was not associated with obtaining non-competitive employment by six-month follow-up in either study group. Based on these findings, an argument could be made that the strength of one’s job interview skills are less relevant when it comes to obtaining non-competitive employment. Thus, future research might consider using non-competitive employment as a marker of divergent validity for job interview skills assessments.

### Implications for research and practice

Based on the initial empirical support for the A-MIRS’ structure, reliability, and validity, future research studies might consider using this assessment when evaluating job interview skills of autistic transition-age youth. This recommendation is notable as the literature consists of at least eight job interview interventions (or comprehensive employment readiness packages that include a job interview training component) that are still in the early stages of scientific evaluation and could benefit from a job interview skill assessment with empirically-supported psychometric properties (18, 20–22, 97–100). In addition to its potential for scientific utility, the A-MIRS could also provide special education teachers, vocational rehabilitation counselors, job coaches, and paraprofessionals, among others, with an evidence-informed method to evaluate the job interview skills of their client. This assessment could be used to work with clients to identify their job interviewing strengths and areas where they may consider strengthening their skills.

### Limitations and future directions

The findings should be interpreted while considering study limitations. First, the present study used baseline data from two RCTs and was not intentionally designed to assess the A-MIRS psychometric properties. Thus, the variables used to evaluate the A-MIRS’ validities were limited in scope. In the future, variables such as the domain scores from the Autism Diagnostic Interview – Revised (101) or an independent rating of social ability [e.g., social skills performance assessment (53)] could be used to further assess the validity of the A-MIRS. Second, the A-MIRS’ criterion validity had minimal support as the employment history variables were significant or trended towards significance but had magnitudes <0.30. In particular, these magnitudes could be limited due to low rates of current or lifetime employment observed in the sample. Future research on the A-MIRS psychometrics might consider intentionally recruiting participants with more work history. Third, interview skills may be influenced by several factors that were not evaluated in this study. For instance, the amount (or quality) of prior job interview training experiences were not assessed and may be stronger markers of criterion validity. Also, job interview skills may have diminished since the attainment of employment which could explain smaller magnitude correlations observed with prior or current employment. Fourth, our Pre-ETS only group did not obtain competitive employment so we could not evaluate its relationship with post-test A-MIRS as a marker of predictive validity. Fifth, we used neurotypical raters which may have biased their ratings of participants with autism, and the neurotypical identities of most coauthors may have biased our development of the A-MIRS and its anchors. Sixth, our study assessed internalizing and externalizing behaviors along with social challenges using teacher or parent assessments (and not autistic self-reports). Notably, the use of either parent or teacher report has limited reliability (102, 103). Seventh, we did not obtain data on how autistic youth felt about completing the measure (e.g., issues around comfort, burden, perceived appropriateness). However, key autistic young adults and community partners (e.g., parents, teachers) were engaged in developing the A-MIRS content as an adaptation from the original MIRS. Eighth, a participant’s IEP or the SRS-2 were used to identify participants as meeting study criteria for autism. These methods may have limited sensitivity and specificity which could result in false positives regarding the diagnosis of autism. Thus, the use of these measures limits the generalizability of the study results. Future research on the psychometric properties of the A-MIRS would benefit from enrolling participants identified with standardized clinical assessments. Finally, the job interview intrinsic motivation scale has not yet been validated and future research is needed to evaluates its psychometric properties.

## Conclusion

Pre-ETS commonly facilitate job interview training for autistic transition-age youth. However, the field of Pre-ETS does not yet have an interview skills assessment with demonstrated reliability and validity that can be used to elevate services or used in research testing job interview interventions. Thus, this study revealed an 11-item unidimensional measure of job interview skills with strong reliability and initial support for construct, divergent, and predictive validities among autistic transition-age youth. Though notably, the support for convergent and criterion validities were limited. Overall, it appears that the A-MIRS has the potential to be a useful tool for research and evaluating the interviewing abilities of autistic transition-age youth who are involved in Pre-ETS or other transition services.

## Data availability statement

The datasets presented in this study can be found in online repositories. The names of the repository/repositories and accession number(s) can be found at: https://nda.nih.gov/ National Institute of Mental Health Data Archive.

## Ethics statement

The studies involving humans were approved by University of Michigan's Health Sciences and Behavioral Sciences Institutional Review Board (IRB-HSBS) and the Kessler Foundation's Institutional Review Board. The studies were conducted in accordance with the local legislation and institutional requirements. Written informed consent for participation in this study was provided by the participants or their legal guardians/next of kin.

## Author contributions

MS, KS, HG, BR, LW, LB, DT, CB, BS, and MK contributed to conception and design of the study. BR organized the database. MK performed the statistical analysis. MS wrote the first draft of the manuscript. KS, HG, LW, LB, DT, CB, BS, and MK contributed to reviewing, editing, or writing sections of the manuscript. All authors contributed to the article and approved the submitted version.

## Footnotes

^1^ www.simmersion.com
