# Supplementary material for: Psychometric properties of the mock interview rating scale for autistic transition-age youth
Source: Front Psychiatry. 2023 Nov 6;14:1235056. doi: 10.3389/fpsyt.2023.1235056 (PMC10657996; doi:10.3389/fpsyt.2023.1235056)
Supplement: Supplementary file 1 [file Presentation_1.pdf]

Appendix A. Autism Mock Interview Rating Scale Job Scenarios

Wondersmart is a nation-wide superstore looking to hire for a variety of entry-level positions:

Scenario A: Auto Repair

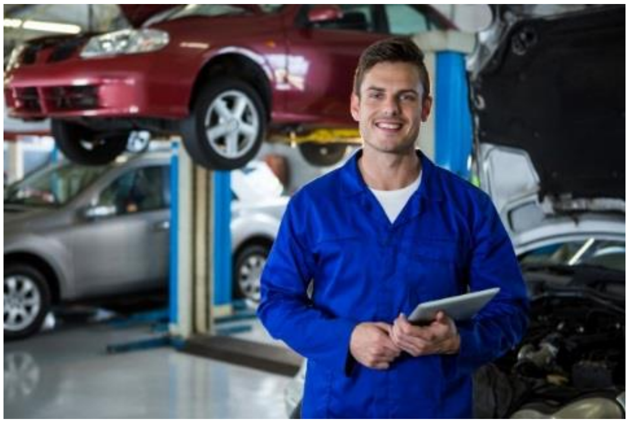

Wondersmart is looking to add motivated Automotive Repair Technicians to their dynamic team! Job responsibilities will include, but are not limited to: performing maintenance and repairs with efficiency in accordance with Wondersmart standards, explaining required repairs in a non-technical manner to customers, and maintaining a clean and organized work area. We offer on-the-job training and a great working environment to progress your career in the automotive industry.

Scenario B: Greeter

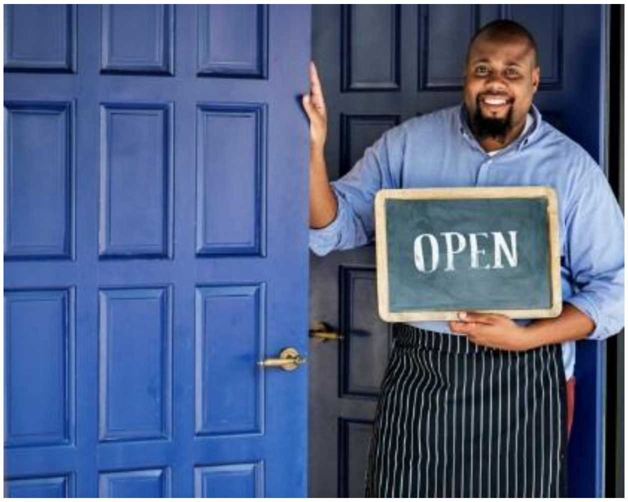

Welcome to Wondersmart! Do you have a friendly attitude and people skills? We are hiring Greeters to politely and happily welcome customers at our Wondersmart entrances. Typical duties include handing out coupons and flyers, welcoming customers, helping with shopping carts, and answering customer questions. You must maintain courtesy and provide exceptional customer services at all times.

Scenario C: Clerical

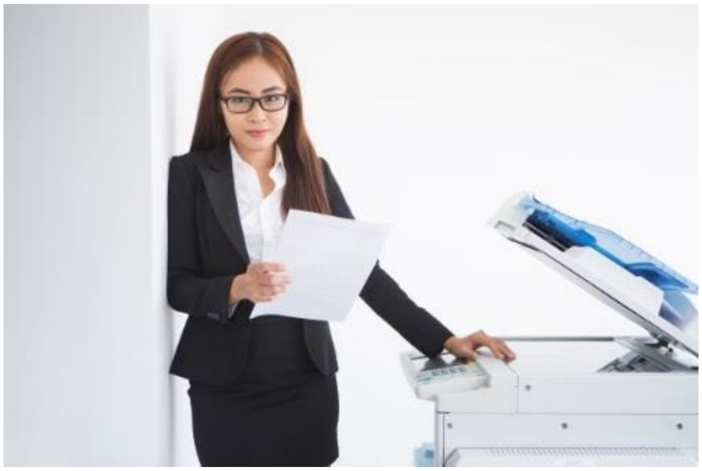

Wondersmart is looking for organized, efficient Office Clerks to perform several clerical and administrative tasks to improve our productivity. An effective Office Clerk will have the capacity to help maintain smooth office operations, including filing of records, sorting and distributing of mail, and answering the phone to take messages or transfer calls to appropriate personnel.

Scenario D: Child Care

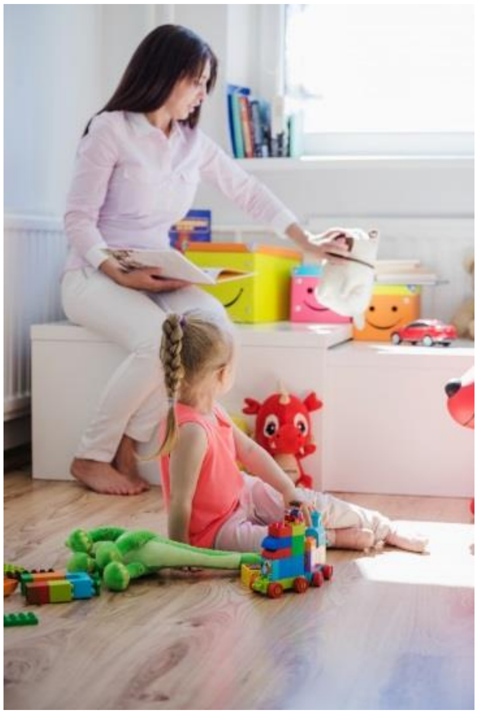

Love kids? Wondersmart is seeking reliable and caring Child Care Assistants who love playing with children and guiding them in their development. Child Care Assistants will care for children under the direction of a Lead Teacher. Duties you would assist the children with include: participating in play activities, reading, painting, drawing, and crafts, and assistance with serving meals and snacks.

Scenario E: Tech Support

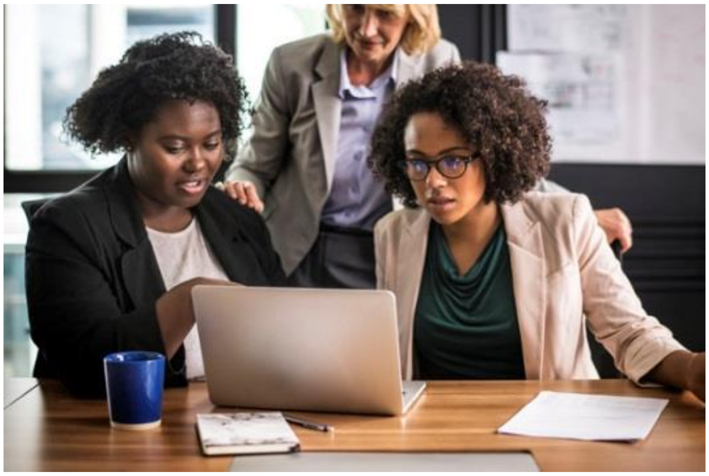

The main role of the Tech Support Specialist is to provide the highest level of support for issues related to computers, printers, wireless devices, servers, etc. The Tech Support Specialist must (1) address support problems reported by Wondersmart customers, team members, and vendors, (2) provide recommendations on software or hardware, and (3) track and inform customers of the status of their requests throughout the process and promptly resolve issues.

Scenario F: Web Developer

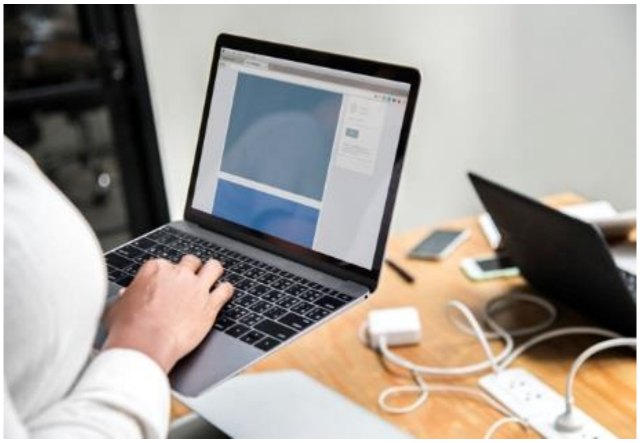

Are you creative and good with computers? Wondersmart is looking for a part-time Web Developer to help redesign and customize the Wondersmart website. Key responsibilities include: assist the Design Manager with projects, design website mockups, review designs with Design Manager, build and launch website, provide ongoing website updates and support.

Scenario G: Stock Clerk

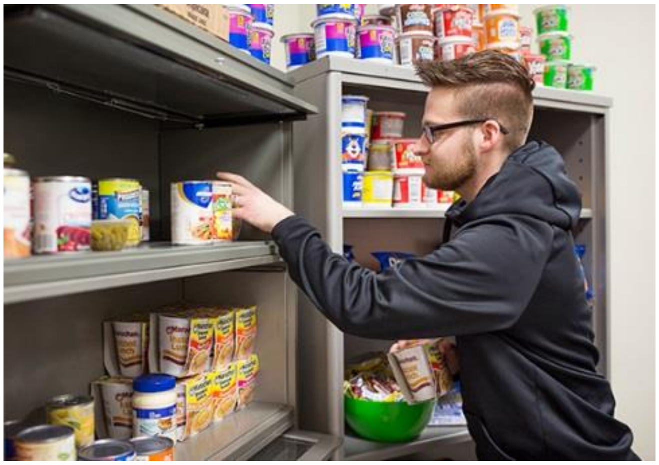

Love keeping things organized? Wondersmart is hiring a detail-oriented Stock Clerk. Responsibilities include receiving and stocking products, including checking expiration dates and rotating products to the front of each display, reporting any discrepancies to a supervisor, maintaining the stock room and keeping it clean and organized, breaking down and recycling empty boxes, and keeping aisles free of debris to ensure a clear path for customers.

Scenario H: Cashier

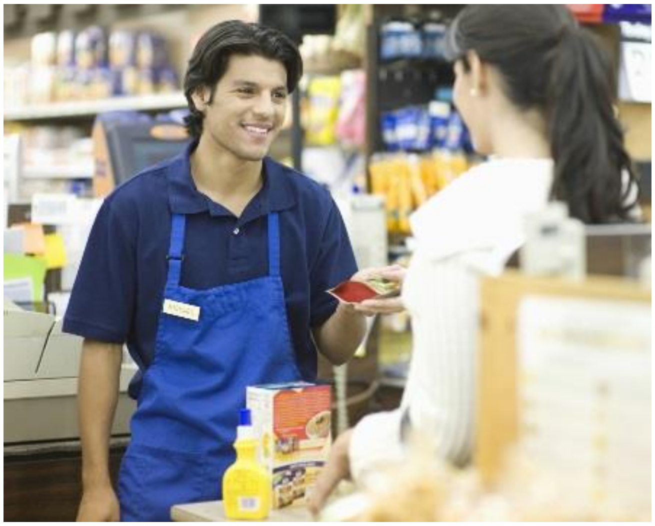

As a Wondersmart Cashier, your main responsibility will include using a cash register to help customer make their purchases. You will be responsible for accepting different methods of payment, while making sure Wondersmart customers experience a friendly and brief checkout. You will be interacting with a high number of customers in a fast-paced setting and making sure we can meet their shopping needs.

Scenario I: Customer Service

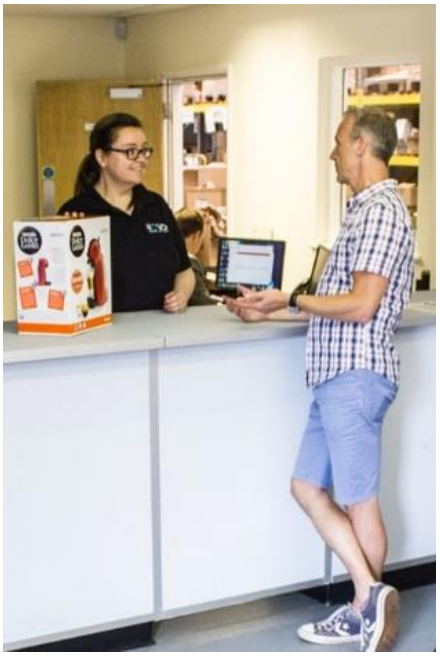

In the role of a Wondersmart Customer Service Representative, your primary responsibility will be to deliver exceptional customer service through friendly and efficient interactions. This will involve demonstrating sincerity, patience, and respect towards customers at all times and expressing gratitude for their patronage. Customer Service Reps must also stay current with present and future Wondersmart sales ads, and communicate special promotions to customers. Must have interpersonal, communication, and customer service skills.

Scenario J: Food Services

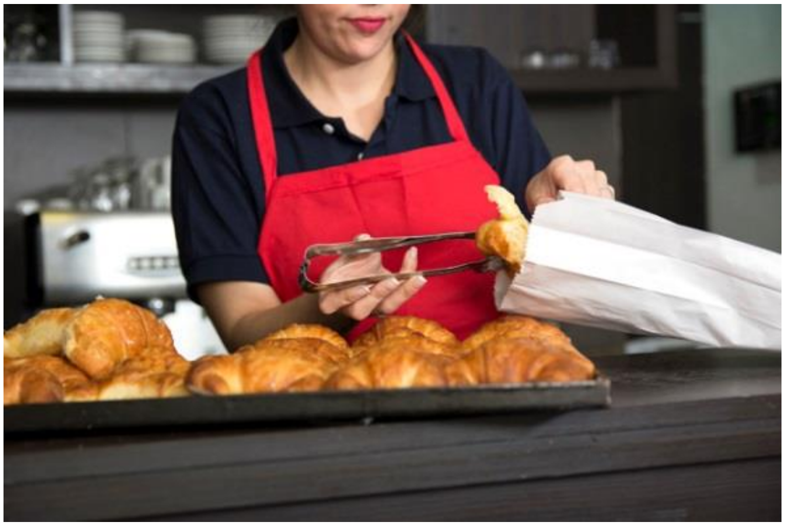

At Wondersmart, our Food Service team is driven by a deep commitment to providing our guests with the finest food and essential items available. As a member of the Food Service team, your primary role will involve warmly welcoming customers, addressing their inquiries and requests with courtesy and professionalism. Additionally, you will be responsible for answering phone calls, taking note of special orders, operating the cash register to process customer transactions, ensuring accurate payment handling, and maintaining equipment safety. It is crucial for all team members to strictly adhere to local, state, and federal health codes and regulations in order to uphold the highest standards of safety and hygiene.

Scenario K: Inventory

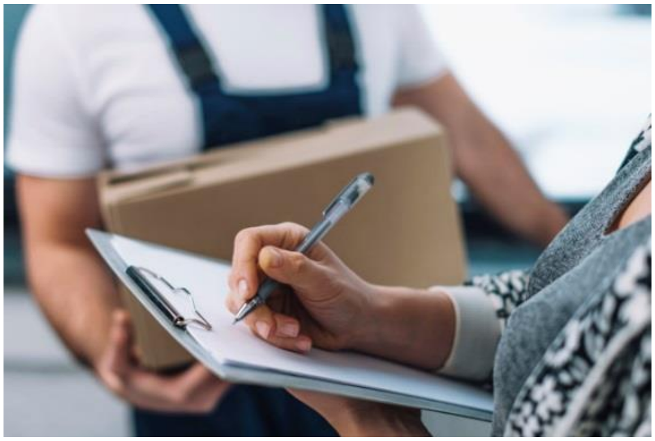

Wondersmart is hiring Inventory Clerks to perform inventory tasks such as physically counting and stocking merchandise and supplies. You will also help with inventory counts and performing re-counts as needed. Attention to detail is a requirement of this position.

Scenario L: Janitorial

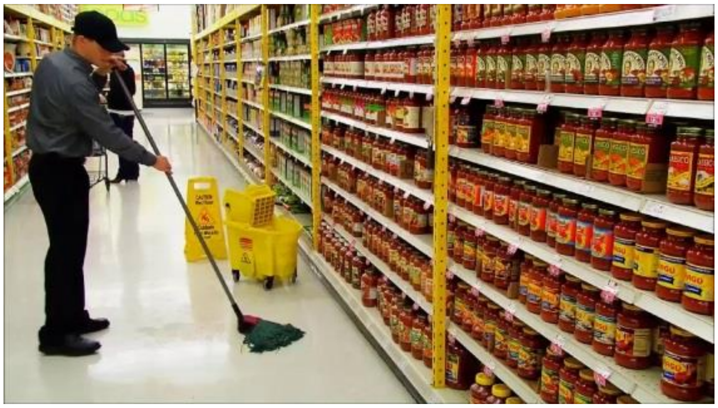

Like to keep things tidy? Wondersmart Janitorial duties consist of sweeping, mopping, garbage removal, putting away and organizing supplies, cleaning display cases, as well as general cleaning. Janitorial staff are also responsible for cleaning up spills and completing spill reports. Must be able to use cleaning equipment safely and follow established procedures.

Scenario M: Maintenance/Grounds

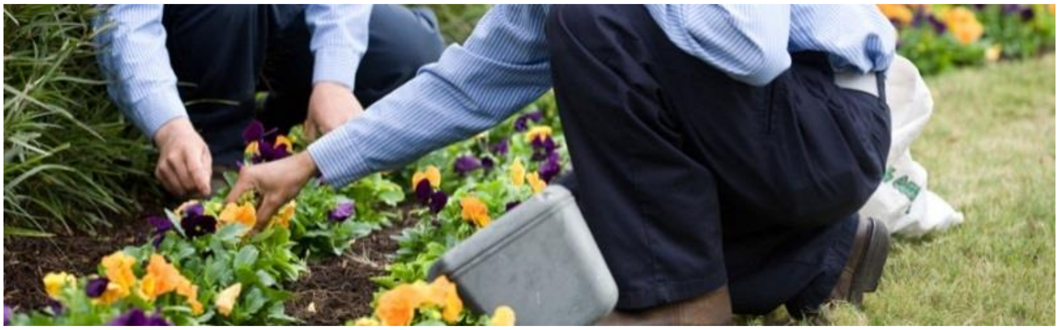

As a Wondersmart Maintenance and Groundskeeper, you will be responsible for operating equipment such as hedge trimmers, leaf blower, and pressure washer, maintaining flower beds, mulching, trimming, weeding, and keeping the parking lots and grounds free of debris.

Scenario N: Security

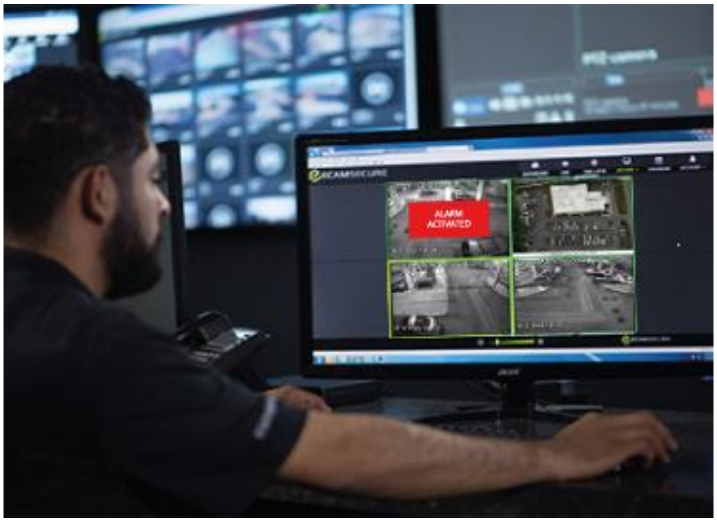

As a Wondersmart Security team member, you will assist the Security Manager with store surveillance using cameras and walking on the sales floor. The Security team also promotes store safety practices, produces and submits timely detailed reports and maintains a good relationship with local law enforcement agencies.

Appendix B. Fidelity Checklist for Job Interview Roleplay

General reminders: The interview questions you ask can be asked in the below order but they don't need to come in any particular order. Instead, there should be a natural flow so feel free to jump around or let the participant guide the conversation. The questions below in red MUST be asked. The rest of the questions you can ask if you have time available. Checkmark all the questions you ask. Interview should only take 20 minutes in total.

Circle which Job Scenario you are doing:

A	*B*	*C*	*D*	*E*	*F*	*G*
H	*I*	*J*	*K*	*L*	*M*	*N*

Script: Hello! Thank you for applying for the ________ position. I have a few questions here for you, so if you are ready let's get started. As we begin, please tell me a little bit about yourself (keep track if this response answers one of the below questions).

- ❑ *What are two of your strengths that will help you work here?*
- ❑ *What are two things a supervisor or teacher has told you to improve? How have you begun to improve them?*
- ❑ *Tell me about your ability to work on more than one project at once.*
- ❑ *Give me an example of a time when you went above and beyond on a task.*
- ❑ *What interests you most about this position?*
- ❑ *Do you have any questions about the job or the company?*
- ❑ *Do you think it's a big deal to show up to work a few minutes late?*
- ❑ *Why is it important to be on-time?*
- ❑ *What type of work environment do you prefer (prompt: on a team or independently)?*
- ❑ *Please tell me what you like about working on a team (or working independently).*
- ❑ *Can you tell me about a time you had a conflict with someone and how you resolved it?*
- ❑ *If you saw a coworker stealing, how would you respond?*
- ❑ *Tell me about a time you had to follow a policy or rule you didn't agree with.*

- ❑ *Tell me about a time you handled a difficult situation (for example, solving a problem).*
- ❑ *Do you have any questions?*

Extra:

- ❑ *How has your past experience prepared you for working at our company?*
- ❑ *What are your career goals?*
- ❑ *What were your responsibilities in your last position?*
- ❑ *How well do you handle stress?*
- ❑ *How would your former coworkers or classmates describe you?*
- ❑ *What would your teachers or supervisors say about you?*
- ❑ *Who was your favorite teacher or supervisor and why?*
- ❑ *Have you ever been on a team where people worked well together? What made it work well or work poorly?*
- ❑ *Give an example where you were able to help someone perform better.*
- ❑ *If you found out your coworker was doing something against company policy, what would you do?*

Complete after interview:

Level of Engagement –

Excellent (5)	(4)	Average(3)	(2)	Poor (1)
• Fully engaged in the interview<br>• Is consistently conversational<br>• Responses are applicable and detailed	• Mostly engaged in the interview<br>• Is conversational most of the time<br>• Responses are somewhat applicable and detailed		• Somewhat disengaged in the interview<br>• Is conversational some of the time<br>• Responses are occasionally short, repetitive and/or uninformative	• Completely disengaged in the interview<br>• Is not conversational<br>• Responses are short, repetitive and/or uninformative
Comments:				
5	4	3	2	1

Appendix C. Autism Mock Interview Rating Scale Anchoring System

Likeliness to be Hired – First Watch

This category is the primary index for assessing whether the participant is likely to be hired for this particular job. Higher scores reflect a likeliness to be hired based on overall interview performance. Lower scores reflect an unlikeliness to be hired.

– Likeliness to be hired – Pre-Test

Very Likely (7)		(5)	Average(4)	(3)		Unlikely (1)
Comments:						
7	6	5	4	3	2	1

Item 1. Being Confident

This category is the primary index of the participant's level of confidence throughout the entire interview. Consider both verbal and nonverbal social cues for signs of comfort/discomfort (tense, sweating, wringing hands, fidgeting, stammering). Participants will score higher if they are prepared, give complete answers, and provide examples. Participants will score lower if they provide partial answers, if their responses require a lot of follow-up clarification, or if they continuously lose their train of thought.

Item 1 – Being confident –

Excellent (7)	(5)	Average(4)	(3)	Poor (1)
• *Casual & relaxed affect*<br>• *Never fidgets*<br>• *Does not lose train of thought*<br>• *Gives complete answers most of the time*<br>• *Provides examples most of the time*<br>• *Consistently responds confidently*	• *Mixed affect, majority relaxed*<br>• *Fidgets less than half of the time*<br>• *Seldom loses train of thought*<br>• *Gives complete answers some of the time*<br>• *Provides examples some of the time*<br>• *Responds confidently most of the time*		• *Mixed affect, majority anxious*<br>• *Fidgets more than half of the time*<br>• *Sometimes loses train of thought*<br>• *Gives a combination of complete and partial answers*<br>• *Rarely provides examples*<br>• *Responds confidently some of the time*	• *Tense/anxious affect*<br>• *Constantly fidgeting*<br>• *Continuously loses train of thought*<br>• *Gives partial answers that require follow-up*<br>• *Never provides examples*<br>• *Does not respond confidently*
Comments:				

7	6	5	4	3	2	1

Item 2. Being Positive

This category is the primary index for assessing whether participants share information about themselves in a positive way. Participants will score higher if they provide multiple examples of being flexible to change, and reframing prior experiences in a positive light suggesting that they learned from the experience. Participants will score lower if they share prior experiences of being inflexible, or that come across negatively (e.g., reflecting on past failures without reframing positively).

Item 2 – Being positive –

Excellent (7)		(5)	Average(4)	(3)		Poor (1)
• *Shares positive attributes with at least one example*<br>• *Displays a positive mindset by emphasizing past lessons learned*<br>• *Shares multiple examples of being flexible/adaptable*		• *Shares positive attributes, without examples*<br>• *Displays a positive mindset*<br>• *Shares one example of being flexible/adaptable*		• *Does not share positive attributes*<br>• *Makes statements that display a negative mindset*<br>• *Shares one example of being inflexible/unadaptable*		• *Shares negative attributes with examples*<br>• *Reframes positive situations in a negative way*<br>• *Shares multiple examples of being inflexible/unadaptable*
Comments:						
7	6	5	4	3	2	1

Item 3. Being Professional

This category is the primary index of whether the participant carries themselves in a professional manner. Higher scores will reflect participants who are respectful, polite, and provide work-related responses. Lower scores will reflect participants who overshare personal information, discuss inappropriate social topics (e.g., politics, religion, partying), and come across as too casual.

Item 3 – Being professional –

Excellent (7)	(5)	Average(4)	(3)	Poor (1)
• *Consistently provides specific work-related responses*<br>• *Provides polite responses throughout the interview*<br>• *Avoids discussing or joking about inappropriate topics*<br>• *Discloses personal information in an appropriate, productive manner*	• *Provides work-related responses most of the time*<br>• *Is polite throughout most of the interview*<br>• *Avoids inappropriate topics most of the time*<br>• *Productive self-disclosure is present but not consistent*		• *Provides work-related responses some of the time*<br>• *Displays some signs of impoliteness*<br>• *Discusses inappropriate topics some of the time*<br>• *Self-disclosure is hesitant*	• *Does not provide work-related responses*<br>• *Is not polite during the interview*<br>• *Discusses or jokes about inappropriate topics*<br>• *Discloses inappropriate personal information (oversharing)*

Comments:

7	6	5	4	3	2	1

<u>Item 4. Showing Interest</u>

This category is the primary index of whether the participant sounds interested in the position. Higher scores will reflect participants who ask several thoughtful questions during the interview, reflect on job duties and how personal strengths can be applied to these duties, and asks about next steps for the employer to make a decision. Lower scores reflect an ‘any job will do’ attitude, or inaccurate citing of the job description.

Item 4 – Showing interest –

Excellent (7)	(5)	Average(4)	(3)	Poor (1)
• *Explicitly displays enthusiasm for the job*<br>• *Asks multiple questions related to job*<br>• *Cites job description in detail*<br>• *Asks about next steps **<u>and</u>** verifies follow-up details*	• *Displays some enthusiasm for the job*<br>• *Asks at least one question related to job*<br>• *Generally cites the job description*<br>• *Asks about next steps **<u>or</u>** verifies follow-up details*		• *Displays little enthusiasm for the job*<br>• *Asks question, but does not express interest in position*<br>• *Does not cite job description*<br>• *Does not ask about next steps or verify follow-up details*	• *Displays no enthusiasm for the job*<br>• *Asks no questions*<br>• *Inaccurately cites job description*<br>• *Disregards or dismisses comments about next steps or follow-up details*
Comments:				

7	6	5	4	3	2	1

Item 5. Being Honest

This category is the primary index of whether the participant sounds honest. Higher scores reflect a participant who comes across as believable, with clear, consistent, well-articulated answers. Participants will receive a lower score if a dishonest statement is made or if they would fail to follow a rule or report a coworker caught stealing.

Item 5 – Being honest –

Excellent (7)	(5)	Average(4)	(3)	Poor (1)
• *Presents themselves as trustworthy throughout entirety of interview*<br>• *Makes statements about ethical work practice with examples*<br>• *Does not provide any answers or examples that are contradicting*<br>• *States they would report theft*<br>• *Makes statement about following a policy or rule, with example*	• *Presents themselves as trustworthy throughout most of the interview*<br>• *States that they are honest and ethical without providing examples*<br>• *Very few answers or examples that are contradicting*<br>• *Hesitates about stating they would report theft*<br>• *Alludes to following a policy or rule, without example*		• *Presents themselves as trustworthy throughout some of the interview*<br>• *Alludes to unethical work practice without providing examples*<br>• *Provides some answers or examples that contradict one another*<br>• *States they would not report theft*<br>• *Answers related to following a policy or rule are ambiguous or avoidant*	• *Presents themselves as untrustworthy throughout entirety of the interview*<br>• *Provides examples of participating in dishonest or unethical work practices*<br>• *Frequently provides answers or examples that contradict one another*<br>• *States it is okay to steal*<br>• *Makes statement related to going against a policy or rule*
Comments:				

7	6	5	4	3	2	1

Item 6. Being dependable or hardworking

This category is the primary index for assessing whether the participant comes across as a hard worker. Participants will be scored higher if they self-identify that they are a hard worker and use specific examples to support this claim. During these examples, participants indicate that they are self-motivated, stay on-task, make work a priority, and give examples of being efficient. Participants will score lower if they mention they are late to work or miss work frequently.

Item 6 – Being dependable or hardworking –

Excellent (7)		(5)	Average(4)	(3)		Poor (1)
• *Describes self as hard worker, with examples*<br>• *Provides multiple examples of staying on task & prioritizing work*<br>• *Provides example of ability to multitask*<br>• *Answers "above & beyond" question with example*<br>• *Expresses importance of being on-time*		• *Describes self as hard worker with no examples*<br>• *Provides one quality example of staying on task & prioritizing work*<br>• *Describes self as good at multitasking, without examples*<br>• *Alludes to going "above & beyond", without examples*<br>• *Expresses importance of being on-time, with qualifier (e.g., only during busy times, etc.)*		• *Provides some indication of laziness or unreliability, without examples*<br>• *Alludes to tardiness, missing work, or avoiding responsibilities*<br>• *Describes self as average at multitasking, without examples*<br>• *Does not go "above & beyond"*<br>• *Expresses indifference to being on-time*		• *Provides direct or indirect example of laziness or unreliability*<br>• *Provides examples of showing up late, missing work, or avoiding responsibilities*<br>• *Describes self as not able to multitask*<br>• *Does less than expected on-the-job*<br>• *States that it is not important to be on-time*
Comments:						
7	6	5	4	3	2	1

Item 7. Working Well With Others

This category is the primary index for assessing whether the participant sounds easy to work with. Participants will score higher if they state they work well on a team setting and provide convincing examples. They will also score high if they indicate they take direction well, and get along with others. Participants will score lower if they complain about former co-workers or supervisors, reflect on prior unresolved conflicts, or complain about prior jobs in general.

Item 7 – Working well with others –

Excellent (7)		(5)	Average(4)	(3)		Poor (1)
• *Discusses being easy to work with while providing multiple examples*<br>• *Makes statement relating to: getting along with teammates, helping customers, listening and taking direction well*<br>• *Able to successfully work on a team*<br>• *Able to resolve conflicts*<br>• *Provides multiple examples of initiating great teamwork*		• *Generally sounds easy to work with & provides at least one example*<br>• *Seems flexible and likely easy to work with*<br>• *Somewhat able to work on a team*<br>• *Somewhat able to resolve conflicts*<br>• *Provides one example of initiating great teamwork*		• *Generally sounds easy to work with, but shares some signs of difficulty*<br>• *Makes statements that allude to being difficult to work with such as poor listening skills, being inflexible or unwilling to follow instructions*<br>• *Has some difficulty working on a team*<br>• *Able to resolve conflicts with assistance*<br>• *Provides no examples of initiating teamwork*		• *Discusses and provides examples of being difficult to work with*<br>• *Explicitly discusses topics related to: bad-mouthing coworkers/boss, having conflict with others, complaining, difficulty compromising*<br>• *Not able to work on a team*<br>• *Has difficulty resolving conflicts*<br>• *Provides an example of initiating poor teamwork*
Comments:						
7	6	5	4	3	2	1

<u>Item 8. Sharing Strengths and Skills</u>

This category is the primary index for assessing whether the participant shares their strengths and skills. Participants will score higher if they share multiple examples of their strengths and skills, and talk about how they use those strengths or skills on the job. Participants will score lower if they do not share relevant strengths or skills, or focus on their weaknesses.

Item 8 – Sharing strengths and skills –

Excellent (7)	(5)	Average(4)	(3)	Poor (1)
• *Provides multiple examples of strengths or skills related to work, school, or home*<br>• *Explicitly states detailed career goals*<br>• *Shares multiple examples of strengths and skills in action*	• *Provides one example of strengths or skills related to work, school, or home*<br>• *Briefly or vaguely states career goals*<br>• *Shares one example of strengths and skills in action*		• *Discusses strengths and skills, but does not share an example related to work, school, or home*<br>• *Does not discuss career goals*<br>• *Does not share example of strengths and skills in action*	• *Does not discuss strengths or skills*<br>• *Makes statement that alludes to not having career goals*<br>• *Shares an example of an undesirable skill or weakness*
Comments:				

7	6	5	4	3	2	1

Item 9. Sharing Past Experiences

This category is the primary index for assessing whether the participant shares their past experiences. Participants will score higher if they share multiple examples of their past experiences and how those experiences will inform their work at this job. Participants will score lower if they do not share relevant past experiences.

Item 9 – Sharing past experiences –

Excellent (7)	(5)	Average(4)	(3)	Poor (1)
• *Shares multiple examples of past experiences with a positive frame*<br>• *Provides multiple details of previous responsibilities at home, work, or school*<br>• *Discusses experience with previous teacher or supervisor in detail*	• *Shares one example of a past experience with a positive frame*<br>• *Provides some details of previous responsibilities at home, work, or school*<br>• *Briefly discusses experience with previous teacher or supervisor*		• *Does not share an example of a past experience*<br>• *Does not provide details on previous responsibilities at home, work, or school*<br>• *Alludes to experience with previous teacher or supervisor*	• *Shares past experience with a negative frame*<br>• *Had no previous responsibilities at home, work, or school*<br>• *Does not discuss experience with previous teacher or supervisor*
Comments:				

7	6	5	4	3	2	1

Item 10. Sharing Past Limitations

This category is the primary index for assessing whether the participant shares past limitations. Participants will score higher if they share multiple examples of how they have overcome difficult situations, handled stress, or showed resilience or perseverance. Participants will score lower if they are unable to handle stress, or focus on limitations and how those limitations may prevent them from doing a job.

Item 10 – Sharing past limitations –

Excellent (7)	(5)	Average(4)	(3)	Poor (1)
• *Shares two personal improvements they have been working on*<br>• *Shares multiple examples of handling difficult situations*<br>• *Provides multiple examples of handling stress well*	• *Shares one personal improvement they have been working on*<br>• *Shares one example of handling a difficult situation*<br>• *Provides one example of handling stress well*		• *Does not share any personal improvements they have been working on*<br>• *Does not share any examples of handling difficult situations*<br>• *Does not provide an example of handling stress well*	• *Demonstrates or expresses refusal to work on any personal improvements*<br>• *Demonstrates or discusses inability to handle difficult situations*<br>• *Discusses or displays inability to handle stress well*

Comments:

7	6	5	4	3	2	1

Item 11. Overall Rapport

This category is the primary index for assessing the overall rapport established between the participant and the HR representative. Higher scores reflect a smooth interaction where the participant was able to convey strong personal skills such as active listening, appropriate eye contact, friendliness, and expressiveness. Lower scores will reflect disorganized conversation, poor eye contact, lack of friendliness, and inattentiveness.

Item 11 – Overall rapport –

Excellent (7)	(5)	Average(4)	(3)	Poor (1)
• *Displays positive affect & demeanor most of the time*<br>• *Displays consistent, appropriate use of eye contact*<br>• *Consistently nods and displays attentiveness*<br>• *Initiates positive interactions at beginning & end of interview*	• *Displays positive affect and demeanor some of the time*<br>• *Displays appropriate use of eye contact most of the time*<br>• *Frequently nods and displays attentiveness*<br>• *Engages in positive interactions at beginning & end of interview*		• *Displays negative affect & demeanor some of the time*<br>• *Displays appropriate use of eye contact some of the time*<br>• *Frequently displays inattentiveness*<br>• *Engages in negative interactions at beginning & end of interview*	• *Displays negative affect & demeanor most of the time*<br>• *Does not display appropriate use of eye contact*<br>• *Appears inattentive most of the time*<br>• *Facilitates negative interactions at beginning & end of interview*
Comments:				

7	6	5	4	3	2	1

Likeliness to be Hired – Second Watch

This category is the primary index for assessing whether the participant is likely to be hired for this particular job. Higher scores reflect a likeliness to be hired based on overall interview performance. Lower scores reflect an unlikeliness to be hired.

– Likeliness to be hired – Post-test

Very Likely (7)	(5)	Average(4)	(3)	Unlikely (1)
Comments:				

7	6	5	4	3	2	1

Appendix D. Autism Mock Interview Rating Scale Raw Score Conversion Table

A-MIRS Raw Summed Score	T Score	T Score SE	A-MIRS Raw Summed Score	T Score	T Score SE
11	-16.30	14.69	48	50.70	2.84
12	-6.20	8.31	49	51.73	2.85
13	0.07	6.13	50	52.76	2.87
14	4.05	5.20	51	53.80	2.89
15	7.10	4.67	52	54.87	2.91
16	9.63	4.32	53	55.95	2.94
17	11.84	4.07	54	57.05	2.97
18	13.84	3.89	55	58.18	3.01
19	15.68	3.75	56	59.33	3.04
20	17.40	3.64	57	60.51	3.08
21	19.03	3.56	58	61.72	3.12
22	20.59	3.48	59	62.97	3.17
23	22.09	3.42	60	64.26	3.22
24	23.54	3.36	61	65.58	3.27
25	24.95	3.32	62	66.95	3.32
26	26.32	3.27	63	68.36	3.38
27	27.65	3.23	64	69.82	3.44
28	28.94	3.19	65	71.34	3.50
29	30.21	3.15	66	72.92	3.57
30	31.45	3.12	67	74.56	3.65
31	32.66	3.08	68	76.28	3.74
32	33.85	3.05	69	78.09	3.84
33	35.01	3.02	70	80.01	3.97
34	36.15	2.99	71	82.08	4.14
35	37.27	2.97	72	84.35	4.36
36	38.37	2.94	73	86.92	4.69
37	39.45	2.92	74	89.98	5.20
38	40.52	2.90	75	93.94	6.10
39	41.57	2.88	76	100.15	8.27
40	42.61	2.86	77	110.19	14.67
41	43.63	2.85			
42	44.65	2.84			
43	45.66	2.83			
44	46.67	2.82			
45	47.68	2.82			
46	48.68	2.83			
47	49.69	2.83			
